# Akivo Lenzner, 1927–2012

**DOI:** 10.3402/mehd.v23i0.19137

**Published:** 2012-08-17

**Authors:** M. Mikelsaar

**Affiliations:** Department of Microbiology, Medical Faculty, University of Tartu, Estonia

Akivo Lenzner, an Estonian professor emeritus of the University of Tartu since 1993 and a well-known researcher of microbial ecology, passed away on 27th of April 2012 at the age of 85. Akivo Lenzner was of Jewish origin, was born in Tartu, and graduated from a high-level Estonian language gymnasium. His father Aron Lenzner had served as a doctor in the Estonian first independence war in 1918–1920. However, after graduating *cum laude* in 1951 from the Medical Faculty of the University of Tartu, Akivo Lenzner had to serve in the Soviet Army for 9 years as a microbiologist. Still, during that time, he succeeded to complete his thesis on tuberculosis meningitis for Cand. Medical Sciences and returned to Tartu in 1960. He got the Doctor of Sciences degree in 1973 and was elected as Professor of Microbiology in 1974. He led the Institute of Microbiology of the University of Tartu for more than 30 years. He taught microbiology to all medical doctors of Estonia for a very long time. Under his supervision, 14 theses of Cand. Medical Sciences were prepared. His research was closely associated with human microflora, particularly vaginal lactobacilli, with respect to health and disease. He has authored more than hundred publications, mainly in the Russian language.

Particularly, professor Akivo Lenzner played a very important role in pioneering the microbial ecology investigations in Estonia and widening these to the large territory of the previous Soviet Union. Several researchers of the Soviet Union got their first training in microbial ecology of gut and vagina in Tartu. The regular research on the lactobacilli of Russian astronauts was remarkably performed under his supervision at the University of Tartu. The first preparations of indigenous lactobacilli strains were done for long-term flights of several astronauts to prevent their imbalance of microbiota. Unfortunately, only during the conferences of Eastern Block countries, could he disseminate the results of his team to the international research community. The studies were accepted very well in Greifswald, Potsdam-Rehbrücke, Rostock, and Prague. In 1996, Akivo Lenzner was elected as an academician of the Medico-Technical Science Academy of Russia for his great impact and success in the field of human microbial ecology.

In 2008 professor Boris Shenderov had written: ‘In former USSR there were some known scientific groups working out a problem of human microbial ecology. One of the most active and influential was the Estonian group of microecologists led by professor A.A. Lenzner. Estonian scientists were among the first in the Soviet Union who had made the deep investigations of infant and children intestinal microbial ecology, suggested original ideas connected with understanding of immune and biochemical mechanisms of colonization resistance, worked out obvious and simple method of bacterium adhesive activity evaluation with human erythrocytes, isolated the first strains of potential probiotic lactobacilli and so on … Some strains selected in Estonia at that time were used later as base for industrial production of Soviet probiotics’.

The research elaborated by professor Lezner has been successfully proceeded with his previous coworkers both in Estonia and in some other countries, including Russia. The middle aged community of medical doctors and microbiologists remember professor Akivo Lenznerit as a brilliant, energetic, and always well-accepted lecturer and teacher.

*M. Mikelsaar*, Professor Department of Microbiology, Medical Faculty, University of Tartu, Estonia

